# Book Reviews

**Published:** 1823-11

**Authors:** 


### CRITICAL ANALYSIS

OF

### ENGLISH AND FOREIGN LITERATURE

RELATIVE TO THE VARIOUS BRANCHES OF

### Medical Science.

Quæ laudanda forent, et quæ culpanda, vicissim

Illa, prius, cretâ; mox hæc, carbone, *notamus.*—PERSIUS.

### DIVISION I.

### ENGLISH.

ART. I.*—*An Exposition of the Principles of Pathology, and of the Treatment of Diseases.* By DANIEL PRING, M.D. Member of the Royal College of Surgeons, London.—8vo. pp. 512. Underwoods, London, 1823.

[Concluded from page 328.]

HERE we pass over about forty pages of rather minute discussion, which, although not devoid of interest, leads to no very tangible conclusions, or, at least, to none which can well be embodied in the condensed form required in our analysis. The subject, at page 203, turns upon the merits of the treatment suggested by the doctrine of determination. If, indeed, we admit congestion of blood in some given seat as the exciting cause of all diseases, the system of therapeutics would become as simple as (although very different from) that of BROWN, already alluded to. In such case, it would only be necessary to diminish the quantity of blood; and by this means the patient must, of necessity, be sooner or later cured,—unless previously killed by the remedy. But as, we presume, our readers agree with us that determination is neither an universal cause or effect of disease, we proceed to inquire into the circumstances under which its presence is distinctly marked, and the means by which it may be best removed. Determination of blood is more distinctly marked in inflammation than any other disease, and yet it is obvious that there are cases in which no abstraction of blood, to whatever extent it may be carried, is capable of removing this condition; while, on the other hand, it occasionally happens that a treatment directly opposite,—namely, that which increases the flow of blood to the inflamed part, is effectual in its curative agency: such as mercury in inflammation of the liver. This, however, cannot be regarded as militating

* We regret that the length of the article on "Puerperal Fever," has rendered it necessary to postpone its insertion till next month, as we are desirous of giving it without farther subdivision.

against the obvious and acknowledged efficiency of bleeding in the removal of inflammation, although it is the opinion of our author (in which, to a certain extent, we concur,) that blood-letting has been too much held up as the *unicum remedium* in all the forms of inflammatory action. Dr. Pring informs us that he is more in the habit of trusting to other measures; and we have observed throughout that purging and nauseating remedies are generally recommended, in preference to more direct forms of depletion. He even mentions having treated one of the severest cases of pneumonia he ever met with by active purging and nauseating doses of squill and antimony, without abstracting one ounce of blood. This treatment resembles that which is in vogue among many continental physicians, except that more was trusted to the purging, and less to the tartarized antimony. Now, so far as the practice differs, we are disposed to give the preference to the nauseating plan of the Italians; purging having appeared to us of very doubtful efficacy in inflammatory affections of the chest. We would not, however, have our readers misunderstand Dr. Pring's opinions so far as to suppose that he advises the omission of venesection, but merely urges its use with more caution and attention to circumstances than is usually adopted. "I have commonly found, (says he, page 207,) that two bleedings of twenty ounces each, within the first thirty hours, with perhaps a bleeding of eight ounces on the third day, and two or three smaller ones of five or six ounces in the course of the disease, has done all that was to be expected from blood-letting."

In some cases of bronchitis, where the symptoms did not yield to the more usual means, Dr. Pring succeeded in removing the disease by exciting salivation; and has likewise tried the same mercurial treatment in various forms of phthisis pulmonalis, sometimes with the effect of suspending for a time, but never of permanently curing, the complaint in any case of tubercular consumption. On the other hand, he has known ulceration of the lungs, resulting from chronic inflammation, perfectly removed by mercury, although it had existed many weeks, with colliquative sweats, pain in the chest, and purulent expectoration. It appears, however, according to his account, to be very difficult to affect the salivary glands in these complaints; and, as the mercury nevertheless quickens the circulation, increased action is frequently produced in the organs which are already the seat of disease, and the death of the patient proportionally hastened. The too-free use of the lancet is likewise deprecated in phthisis and in rheumatism: in both of these diseases we believe copious bleeding to be highly prejudicial. But we cannot agree with our author when he informs us that, in peritonitis, he has "trusted also much more to purgatives than to bleeding;" notwithstanding that he adds, "out of many cases, under every variety of circumstances, I never met with one which terminated otherwise than favourably." (p. 218.) Regarding the principal indication in peritonitis to consist in removing the constipation of the bowels, our author's practice consists in giving six or eight grains of calomel, followed by a black dose containing jalap, and repeating them as often as they are rejected, till such portion is retained as induces the bowels to act. This object (viz. purging) once effected, he informs us that he has never found bleeding necessary *afterwards*: our own experience in this respect does not correspond with that of Dr. Pring. In phrenitis from local injury, the advantages of blood-letting are admitted as very suspicious; in puerperal mania it is said to be less marked, and still less in ordinary insanity.

The subject of apoplexy receives a considerable share of attention, and numerous cases are related to show the frequent insufficiency of bleeding either to cure or prevent an apoplectic seizure.

"Blood-letting, as a measure of prevention where the tendency to apoplexy prevails, appears liable to the following objections:—1st, that the loss of blood disposes frequently to a more rapid formation of this fluid, of which there is, I believe, of course, with many exceptions, abundant testimony; 2d, frequent and copious bleeding often produces determination to the head, or increases it where it before existed; 3d, there are states of the subjects disposed to apoplexy, in which any change, operating as a cause of excitation, may induce the effect of the tendency, which seems to be resisted only so long as some remains of an habitual balance of functions continue to counteract it; 4th, frequent bleeding increases the proportion of serum in the blood, and, under ordinary states of this fluid, tends to weaken the coagulation of the crassamentum; the muscular or tonic power of the arteries, as is indicated by some cases, bears a proportion to the coagulating power of the blood, and the diminution of this power may concur in exchanging mere determination for sanguineous apoplexy. The force of these objections is by no means insuperable: they may, however, be borne in mind, and their opposition is rather to superfluous or unnecessary bleedings, than to those in which the propriety of the measure is fully confirmed by the hazard of a much worse alternative.

"Blood-letting, in apoplexy, as a means of cure, is indicated both by principle and experience to a certain extent. But it is suggested by many results that, although bleeding to a certain extent may relieve the vessels, and tend to produce a more equal circulation, an excessive loss of blood is commonly itself productive of an irregular circulation, and will therefore tend to increase it where it exists; and perhaps will also concur with the tendency of the disease to a rupture of vessels, by diminishing the power which these structures possess of resisting the impulse of the blood." (p. 235—237.)

At page 239, our author informs us that he has sometimes suspected apoplexy to be occasioned by congestion of vessels merely, without any extravasation; but he does not seem quite satisfied on this head. We ourselves are decidedly of this opinion, and beg to submit the following case in point. A gentleman of full habit, short neck, florid countenance, and convivial disposition, had frequent feelings about his head, which kept him in constant apprehension of suffering more seriously, but did not induce him to live less freely. After a time he became affected with symptoms indicative of pressure about the brain, sometimes assuming the more marked character of coma, which lasted several weeks, and was at length removed by copious purging. From this state he entirely recovered, and about a year after made a very argumentative and animated speech at a public meeting in London: when he sat down, he was observed to lean forwards, and he did not answer a question put to him by his neighbour;—he was quite dead. His head was examined by an eminent surgeon in the city, but neither extravasation nor any kind of effusion was found; there was no disease of the heart or great vessels. We do not relate this case from regarding it as very uncommon, but in answer to Dr. Pring, who says, "the proof of apoplexy from this cause (mere congestion) would be furnished by the results of a careful examination of the brain of a person who died soon after an attack, in which this state of the brain was supposed to have obtained."

Our author was formerly in the habit of bleeding in epilepsy, but was discouraged by some inconveniences with which he found the operation attended; and, indeed, the struggling of the patient has always appeared to us to render venesection extremely hazardous during any convulsive fit.

Dr. Pring insists very strongly, and we think judiciously, against the system of copious and long-continued depletion, on the principle of its increasing the irritability of the heart, and increasing the determination of blood to the head; and we entirely agree with him that cupping, or the application of leeches, in those disorders of the head called nervous, affords much more essential relief than general bleeding.

In hæmoptysis connected with suspended menstruation, the treatment recommended consists chiefly of purging with aloes and gamboge: the warm bath, and even the hip bath, are regarded as doubtful remedies; as the author has known them sometimes determine to the lungs. We certainly never knew this of the hip bath, which has appeared to us a useful remedy in such cases. On hæmoptysis from other causes, we have some good remarks.

"The pathology of hæmoptysis furnishes two important practical distinctions,—namely, hæmoptysis with, and without buffed blood. If blood taken from the arm, in cases of hæmoptysis, is buffed, the local disease tends to rapid disorganization; and, unless arrested by treatment, terminates speedily in consumption. If in such cases the blood is not buffed, there is no immediate danger of consumption: the only danger is from hemorrhage, and this danger is not trifling. The most inconsiderable spitting of blood is always a symptom which requires great attention. If a small vessel of the lungs is ruptured, it proves a disposition in the vessels to this effect, and there is no security but that a large one may next give way. A young man, about twenty years of age, had a pain in his side, and sometimes expectorated a very little blood. He took no notice of these symptoms, and continued his work as usual. One morning he returned from his accustomed labour, and, whilst at breakfast, a copious hemorrhage from the lungs took place: I was immediately sent for, and arrived at the house in a few minutes; but the young man was dead before I got there. The floor was covered with blood, and it appeared that the rapidity with which the effusion of this fluid had taken place had produced suffocation.

"In hæmoptysis with buffed blood, the symptoms cannot be too rapidly subdued by antiphlogistic means. Bleedings of sixteen or twenty ounces, calomel, active saline purgatives, full doses of nitre, squill, ipecacuanha, emetic tartar, &c. and a diet of cold gruel, tea, barley-water, &c. should be employed in the beginning, together with perfect repose. If the case becomes chronic, small bleedings, as a collateral treatment, are advantageous, together with nitre, and constant purging by Epsom salts. I have ample reason for thinking the repetition of large bleedings prejudicial; and a treatment in which blood-letting is made the only remedy, altogether inadequate. I have known all the symptoms cease in these cases, and the blood has no longer exhibited fibrine, when the calomel, which was given in doses of three grains every night, followed by salts and senna the next morning, has affected the gums. I have known also the coagulation of the crassamentum gradually weakened, and the exhibition of fibrine cease, under a treatment by a grain of acetate of lead three times a-day, in combination with a full dose of squill and a small one of digitalis: the saline purgatives, and occasionally calomel, have also been employed at the same time.

"If the blood is not buffed in hæmoptysis, the immediate danger arises from hæmorrhage; but a portion of unabsorbed coagulum will, it is currently believed, at a remote period, form the nucleus of a tubercle: this will be followed by fever, local disorganization; and in this state, as I believe in every other case of consumption, the blood will be found buffed.

"The best treatment of this form of hæmoptysis is also, so far as my experience has gone, to subdue the symptoms by smaller bleedings than in the former cases, by saline purgatives, nitre, squill, ipecacuanha, emetic tartar, &c. and occasionally calomel as a purgative, not frequently repeated. In this case, in which there is no inflammatory action to be subdued, I have no reason to think that any thing would be gained by mercurial affection: on the contrary, it may convert hæmoptysis without fever into that febrile form in which buffed blood would most probably be exhibited. Repose, with a vegetable diet, and that nearly cold, with a temperate atmosphere, are also necessary in this simple form of hæmoptysis. I have known the chest covered constantly with cloths dipped in cold vinegar and water; but, from experience, I have no reason to think this practice beneficial, and, from principle, I should not be inclined to anticipate from it any good results. I once knew a case of pneumonia treated by cold applications to the chest: the boy, who was about fourteen years of age, died; and certainly with a moderation of symptoms, from which I should not apprehend much danger under the ordinary treatment." (p. 256—258.)

Some affections of the heart, dropsy, and diseases of the brain, are next cursorily discussed; but we do not find any thing in this part of the work to detain us.

In concluding the subject of determination of blood, it is remarked that this, when not produced by mechanical obstruction, is in the first instance but an effect, although it may subsequently become a concurring cause of disease; and that blood-letting, therefore, as a therapeutic agent, is defective in principle, but may, and often does, act beneficially by diminishing one of the supporters of disease. But even this limited advantage is not one which we can always obtain, the beneficial effect of bleeding being dependent upon a *curative relation* of the remedy with the disease,—a relation, the knowledge of which can only be acquired by experience.

*Origin of Disease in the Abdominal Viscera.*—This chapter opens with a refutation of the prevailing doctrine, that all, or nearly all, diseases originate in the stomach or liver. The arguments used are similar to those formerly employed by Dr. Pring in his "Indications;" and, as they are intended to disprove the universality, not the frequency, of the abdominal origin of disease, we do not conceive it necessary to recapitulate them at present, but proceed to state the view taken by our author of the various derangements of the digestive organs. The great general division of these is into *exclusive* and *related* diseases. The former admits of no subdivision, and is regarded as uncommon; only three or four cases having occurred to the author, in which disorder of the digestive function appeared totally unconnected with any other disease. Related disease of these organs, on the contrary, is not only of every-day occurrence, but admits of many subdivisions; namely—

"1. Disorder originating in the digestive organs, and producing disease elsewhere, by simple extension.

"2. Disorder originating in the digestive organs, and ceasing by the occurrence of disease elsewhere; which exemplifies a curative relation of other seats, in respect to the digestive organs.

"3. Disorder of the digestive organs originating elsewhere, and exemplifying simple extension of disease.

"4. Disorder of the digestive organs originating elsewhere, and holding a curative relation with respect to its primary seat.

"5. Disorder of the digestive organs, of synchronous origin with disorder elsewhere; at least, disorder of these and other seats, where the succession cannot be defined.

"6. Disorder originating in the digestive organs, and producing disease elsewhere, which is neither one of simple extension nor curative, but in which the secondary re-acts, and increases the primary disorder.

"7. Disorder of the digestive organs, originating elsewhere, which is neither one of simple extension nor curative, but in which the primary disease is exacerbated by that of the digestive organs.

"8. Disorder of primary or secondary seats, including the digestive organs, with or without curative relation, extended to other seats, also with or without curative relation." (p. 294.)

Disorder originating in the digestive organs, and giving rise to disease elsewhere, by simple extension, is illustrated by those local affections which are the consequences of the primary derangement of digestion,—as some kinds of fever, erysipelas, and ulcers.

Disorder originating in the digestive organs, and ceasing under the occurrence of disease in another seat, is exemplified by dyspepsia disappearing on the breaking out of a cutaneous eruption.

Disorder of the digestive functions originating elsewhere, and exemplifying simple extension, are very common, including a large proportion of those cases in which nervous complaints are associated with indigestion. These generally originate in habits of the mind, as study and business, or in the passions. The brain is first disturbed, and the digestive organs become secondarily affected.

Derangement of the digestive organs originating elsewhere, and being curative, or, in the words of our author, having "a certain relation" with the original seat, is illustrated by the abatement of fevers upon the supervention of diarrhœa, or other diseased action assumed by the abdominal organs of secretion.

Disorder of the digestive functions synchronous with that of other parts, occurs in pneumonia, acute rheumatism, &c. where loss of appetite and white tongue come on along with the other symptoms, so that it cannot be said which first made its appearance.

Disorder of digestion producing another disease, which increases the primary one, is exemplified by some kinds of irritable ulcers, which are first brought on by derangement of the chylopoietic viscera, "and then, acting rather by sympathetic irritation than by substituted disease, increase the symptoms in the primary seat."

Secondary disorder of the digestive organs may increase the primary disease. This is illustrated by supposing a gun-shot wound to have become chronic, and the general health to suffer but little in consequence: if, however, from exfoliation or any other cause, the wound should disorder the stomach, such disorder may remain after the exfoliation or other exciting cause has passed away, producing an injurious effect both upon the general health and local injury.

The eighth, and last of Dr. Pring's divisions, is principally met with in complicated forms of disease: "thus, disorder of the head produces disorder of the stomach; disorder of the stomach, abscess, boils, cutaneous eruptions, or diabetes:" the first may be removed or alleviated on the appearance of the second; the second may be similarly influenced by the third; or all three may exist together, and even exacerbate each other.

We come now to Dr. Pring's method of treatment in dyspepsia, which is so entirely at variance with the opinions generally received upon this subject, that, before venturing upon any comment, we request the attention of our readers to the following passages.

"With respect to the usual treatment of dyspepsia, by avoiding all those articles of food which serve to increase the symptoms, I have known many, perhaps some hundreds of cases, so treated, but never knew one cured by it. The disease may be thus palliated; but I doubt if the disposition to it is not increased. There are three errors in this careful and limited selection of the articles of food. The first is, that the stomach can often digest with ease things which people have determined *a priori* it ought not to digest at all. The second is, that, by not keeping the digestive function adequately exercised, it is apt to become capricious, and either loses the inclination or the power to perform its duty. The third is, that, whereas the bile is supposed to be the natural stimulus to the action of the bowels, I have reason to think that the food is not a little concerned in enforcing this action; that people may have griping pains, diarrhœa, tenesmus, &c. with white stools, in which it is presumed there is no bile; that, if persons eat sparingly, the bowels will not act regularly, for want of adequate stimulus derived from this source; that, if they eat abundantly, the stimulus of the ingesta will, to a great extent, contribute to, or produce, regularity of the alvine evacuations.

"I have known a change of diet, from a starving one to one of repletion, followed by regularity of the bowels, so that under the latter regimen the subject did not take an aperient medicine for two years; and under the former he scarcely ever had an evacuation which was not procured by medicine. I knew a young man, twenty-one years of age, who had dyspepsia; he was, however, in other respects, healthy and vigorous; he had a soft pulse, commonly of sixty, and was able to walk ten or fifteen miles in a day without fatigue. His bowels were very torpid, and he was highly nervous. He had been directed to eat only food of a certain quality, and of this food, which was weighed, to take only a few ounces; to drink water, and to procure stools by large doses of the compound camboge pill. I recommended him to leave off his pills, eat as much as he could, and of what he liked, and to drink a quart of strong beer every day. He was persuaded to enter, with great timidity, upon this plan; and in three or four days his trouble was, not that his bowels would not act, but that they acted too much, or too frequently. He was frightened at this effect, and returned to his old regimen, which he persevered in; and in a few months he died of an atrophy: my construction of which event was, that he was starved to death, notwithstanding he possessed a good appetite, and the means of procuring as much food as he liked. It is to be remarked that there was not, in this case, any evidence of diseased mesenteric glands.

"Although, in dyspepsia, the bowels may not act with regularity under any plan of diet, I believe their action will approach nearest to regularity under the regimen of repletion,—that is, of eating and drinking abundantly. This plan will produce occasional inconvenience; but it is attended, so far as my observation goes, with a better general state of health than when the opposite one is adopted. It will every now and then be productive of an exacerbation of the symptoms; and then a purgative, or a short course of purgatives, with a change of diet, will at once be a remedy to the exacerbation, and have the effect of producing afterwards a comparative freedom from the complaint, and an improved state of health. This improvement will, perhaps, last many months; during which time the less the patient thinks about his stomach and bowels the better. Nature might take the matter into her own hands; and, if the bowels are neglected, she will, perhaps, give the patient a slight attack of cholera, which will be followed by all the improvement which usually succeeds to a trifling crisis of a disorder. The benefits of an occasional debauch have been remarked by physicians in almost every age; by which is meant, that getting so drunk as to vomit at the time, and feel very uncomfortable for a day or two afterwards, will sometimes avert other disease, and supersede the use of medicine. This remedy, however, as less objectionable ones may be had, I am far from recommending." (p. 301—304.)

"There are many diseases which we treat with advantage upon something like a principle derived from the effects of habit. I believe dyspepsia is, to a certain extent, one of them. If the eye is so irritable that it will not bear the light, we make it bear laudanum or æther, and afterwards it does not regard the lesser stimulus of light. A similar illustration is afforded by the effects of muscular exercise: a person accustomed to walk fifteen miles a-day, would think but little of walking five. So with respect to the stomach: if it will not bear moderate stimuli, or seems unequal to moderate exercise, we must make it familiar with immoderate stimuli and harder labour, and it will then cease to regard the inconvenience imposed by its habitual duties. The principle is illustrated also by the frequent use of aperient medicines: if the bowels are accustomed to the preternatural stimulus of medicine, they will not act under the natural one of food." (p. 305.)

The first part of the paragraph we have quoted agrees in principle with some remarks which we ventured to make in a recent Number of this Journal.* Indeed, we are satisfied that any one who reflects on the results of his observation, uninfluenced by the authority of medical writers, will readily acknowledge how very rare an occurrence it is to see a case of dyspepsia cured by any of the ordinary methods of treatment. Refraining from such articles of food as are found by experience to disagree with the stomach, and regulating the bowels with aperients, may relieve for the time; but we agree with our author that they leave the disposition to the disease undiminished. That medical men err in the routine practice of forbidding certain articles as a matter of course, and regulating the quantity by some standard of their own, regardless of the appetites of their patients, we are pretty firmly convinced, from observations on others, as well as from personal experience. But, although we are by no means converts to the system of starvation in many forms of dyspepsia, yet we are not prepared to go so far as Dr. Pring, particularly as regards the last paragraph we have given above. To say of the stomach, "that, if it will not bear moderate stimuli," "we must make it familiar with immoderate stimuli," implies that, if a patient cannot digest mutton-chops and madeira, the way to enable him to do so is to prescribe a course of turtle and brandy. If this be not the author's meaning, then we profess not to understand it: if it be his meaning, we regard the doctrine as leading to practice of the most objectionable kind.

Blue-pill, according to the observations of Dr. Pring, increase the symptoms if given in doses of four or five grains every night, but these generally improve when the medicine is discontinued: by very small doses, the disposition to disease is occasionally subverted, if the plan be continued sufficiently long. These effects, however, he regards as by no means invariable from any dose of blue-pill, and seems to entertain a more rational opinion of this wonder-working medicine in indigestion than is generally to be found in practical writers. Purgatives, we are informed, frequently increase the symptoms of the various chronic diseases for which they are administered: "this effect is rather desirable than otherwise, inasmuch as it proves that the remedy *has a relation with the disease;*" and is, consequently, regarded as a motive for their continuance, or even increase. He has not found purgatives successful in dyspepsia with nervous irritability, but eminently so when the disease is accompanied with derangement of the liver, or chronic pain in the side or stomach. Our author speaks rather favourably of caustic issues, when they have become unirritating drains: at first, all the forms of artificial suppuration increase the dyspeptic symptoms.

* Review of Dr. GREGORY's *Theory and Practice of Physic*, No. for June.

On the subject of spontaneous eruptions, we have some good observations:

"The connexion between dyspepsia and diseases of the skin has been before remarked. Those who have written on diseases of the skin, with a great appearance of learning and connoisseurship, have done little more than multiply unnecessary and trivial distinctions, and propose a jargon of barbarous terms, which none but persons of very corrupt taste will take the trouble to remember. I do not, in the works alluded to, remember to have met with any thing like a principle of the pathology or treatment of these diseases. In this place it is necessary only to remark, that, as we have seen in our analysis of the relations of disorder of the digestive organs, diseases of the skin are variously connected with such disorder; so a long-continued treatment by small doses of blue-pill, perhaps with colocynth, or aloes and ipecacuanha, will cure many of them, without recurring to the more powerful agency of calomel, corrosive sublimate, or arsenic; and, as a local application, hartshorn and water, in the proportion of a dram of the former to an ounce of the latter, is almost a specific in many chronic diseases of the skin, attended either with lymphatic or pustular eruptions, whether confined to one spot or extending over a whole limb. The effect of this stimulus is to exchange a peculiar for a common inflammation; and I presume, by the same mode, liquor ammoniæ, liquor potassæ, and turpentine, will cure tinea capitis: the proportion of hartshorn is to be regulated by the irritability of the surface, and either applied constantly or occasionally, according to circumstances. This remedy has long been employed in erysipelas, and I have used it with great success in almost every case of disease of the skin described above, which has fallen under this treatment: if continued after the specific character of the disease seems to be subdued, it appears to irritate and produce troublesome exfoliations of the cuticle." (p. 311—313.)

The sixth chapter, which is the next in the order of our analysis, treats of the *origin of disease in the nerves*, a subject which was formerly discussed by Dr. Pring, with conclusions very different from those contained in the work before us. In his "View of the Relations of the Nervous System," he argued the exclusive agency of the nerves, both with regard to life and to disease; in doing which, he now informs us that he reasoned "badly," and that his opinions were "unphilosophical." Indeed, it is rather amusing to observe the freedom and disregard of all the common affectation of modesty with which the author speaks of his own productions. Referring to his arguments against the nervous origin of diseases, contained in his "Indications, &c." he says, it is desirable "not to be prolix in a refutation of this doctrine, *which is elsewhere as complete as language can express.*" For ourselves, we profess to know too little of the physiology of the nervous system to venture upon discussing its pathology, and therefore must content ourselves with referring such of our readers as patronise the nerves to Dr.

Pring's first work, and those who think differently to his "Indications" and the one before us."

The *Relations of Disease* form the next subject of inquiry, and are treated of in a very scientific and interesting manner. "Diseases are said to be related, when a disease in one seat produces or modifies a disease in another;" and the proof of this influence is derived from succession. But as mere antecedence and consequence do not stand to each other in the relation of cause and effect, so, in tracing the relations of disease, considerable caution is necessary in drawing our conclusions. The author here takes occasion to enter upon the subject of causation, and afterwards proceeds to discuss the relations of disease. Both of these topics were considered at length in the "Indications," which work was so fully analysed in this Journal, as to render any recapitulation superfluous. "Indeed, (says our author,) the whole subject of related, as well as other diseases, has been in this work (viz. "the Indications,") so fully discussed, that very little has remained here to be done, but to extend the analyses formerly indicated, to give a different arrangement to the arguments, or to add to their illustration."

There yet remain three chapters, upon the *General Principles of Pathology*, the *Speculative Doctrines of Therapeutics*, and the *Practical Principles of Therapeutics*. After a careful perusal of these, and some reflection, we have judged it most expedient not to attempt an analysis of this part of the work. We have come to this resolution from considering the length to which this article has already extended, and the difficulty of giving any abridged account of that which is already in a great measure analytic. We could not do so with justice to the author, or satisfaction to ourselves, without devoting to the task a portion of the Journal greater than we believe our readers would think the investigation deserved. We do not say this as undervaluing the importance of these subjects, but from the fact hinted at above, that Dr. Pring is too fond of abstract reasoning to be generally read: in fact, it is not enough to *read* his works, they must be deeply *studied* to be understood;—nor has this always succeeded with ourselves. In making this acknowledgment, we mean not to reflect upon Dr. Pring, for whose talents we have more than once expressed the highest respect; but we trust we do not misconceive the wishes of our readers in selecting, as much as possible, for their perusal such parts of the various works which fall under our review as contain practical and tangible principles, which tend to remove, in some degree, the difficulties of our profession, and are applicable at the bedside of the patient. Such parts of Dr. Pring's "Principles of Pathology" as have appeared to us to fall under this description, we have noticed in our analysis.

The remaining chapters are written in the same spirit of abstract and philosophic inquiry which characterises some of his other works, but they are too general and abstruse for our purpose. Even his "Practical Principles of Therapeutics" are as little *practical*, in the usual acceptation of the term, as it is possible easily to conceive.

In conclusion, we would remark that neither our readers nor the author have reason to regret this arrangement, whether it be well or ill founded, as the leading doctrines of Dr. Pring have been analysed and illustrated, in a manner which we could not hope to have equalled, by our learned predecessor, Dr. HUTCHINSON, who appears to have followed his author through all his reasonings, to have comprehended his views, and acknowledged the justice of his conclusions.

---

ART. II.—*Practical Observations in Surgery.* By HENRY EARLE, F.R.S. Assistant Surgeon to St. Bartholomew's Hospital, and Surgeon to the Foundling.—8vo. pp. 229. Underwood, London, 1823.

ART. III.—*Observations on Fractures of the Neck of the Thigh-bone; being an Appendix to the Work on Dislocations and Fractures of the Joints.* By Sir ASTLEY COOPER, Bart. F.R.S. Surgeon to the King, &c.—4to. pp. 44. Longman and Co. 1823.

WE have never devoted ourselves to our critical labours with so much reluctance as upon the present occasion. Feeling, as we most unfeignedly do, the highest respect and esteem for both the gentlemen whose works appear at the head of this article, we cannot but lament, not their difference of opinion, for we cannot all be expected to think alike upon any subject, but the irritation which the mismanagement of such discussions necessarily tends to produce,—the heart-burnings and jealousies to which it unfortunately may give rise in the respective schools, of which these authors may be considered as the champions,—and, worse than all, the ill effects which professional misunderstandings produce upon the public at large, tending to shake their confidence in the art itself, and throwing something like a reproach upon the character of its members. Our duty is particularly painful, because between such opposite doctrines we must make a choice: we cannot satisfy ourselves or our readers by saying, with Sir Roger de Coverley, "that much may be said on both sides of the question;" we must make up our minds as to the person who is abstractedly in the right, and thereby we must risk giving offence to the party from whom we differ in opinion; but we shall endeavour to clothe our objections in language as dispassionate and soothing as possible, and trust that, if we are so unfortunate as to fail in convincing by our arguments, that at least we shall not offend by the manner in which they are stated. Commencing, then, with Mr.

Earle's work, as first in order, we must be allowed to make one or two prefatory remarks upon its general tone and complexion, and we shall then proceed to discuss its arguments *seriatim.*

In the preface, Mr. E. tells us that, having the misfortune to differ with Sir A. Cooper on the subject of fractures of the neck of the thigh-bone within the capsular ligament, cases in which that gentleman "has stated it as his opinion that perfect union cannot take place," &c. he has thought it therefore incumbent upon him to submit his opinion on the opposite side of the question to the consideration of the profession, in order that they may judge between them. After a very modest allusion to the disadvantages under which he labours, in combatting the opinion of a gentleman enjoying the public confidence and esteem so highly and so deservedly as Sir A. Cooper, our author concludes as follows:

"On an attentive and dispassionate revise of the following work, since the sheets have been printed off, some expressions appear to have escaped me in the warmth and hurry of composition, which may possibly admit of a construction very different from my wishes or intentions. I am anxious, therefore, most distinctly to disclaim the slightest feeling of disrespect towards the author whose work I have reviewed; and, should any passages appear to the reader as too strongly expressed, I must entreat his indulgence in referring them to the ardour of a person writing on a subject which has occupied much of his attention, who was anxious to establish opinions which he has long entertained, and to introduce a practice which he hopes will be found beneficial."

Here we cannot but lament that Mr. Earle did not alter expressions, the tendency of which he evidently must have seen. We are perfectly convinced, that to the ardour and zeal of an author combatting what he conceives to be erroneous doctrines, must alone be attributed the turn of one or two phrases that have escaped his pen, and which, we fear, have given rise to much of the acrimony and irritation that appears in Sir Astley's reply; and it is to be lamented that they were not either struck out or modified before the work was sent to press. This observation we are induced to make, because we have heard these expressions quoted and mentioned as evidences of an hostile spirit in the writer; and we therefore most unequivocally contradict such assertions, because we *know* that Mr. Earle is incapable of feeling such a spirit, and that professional zeal, and a conviction that he was performing a duty, have alone led him to step forward in the discussion of this question.

The work itself is written in a clear and perspicuous style, and commences with a concise description of the anatomy of the thigh-bone in the adult, and which we should not stop to notice, were it not that the paragraph which relates to the description of the situation of the ligamentum teres has given occasion to a criticism on the part of Sir A. Cooper. "The head of the bone (says Mr. Earle, p. 2,) is covered in the recent state by cartilage, excepting at its centre, where there is a depression for the interarticular, or round ligament." This statement Sir Astley contradicts; and, in fact, it must be admitted that the insertion of the ligamentum teres is not precisely in the centre, but a few lines from it. Nevertheless, we cannot but consider this criticism as a little captious, seeing that the usual description is that which Mr. Earle has given, and that the very trifling deviation from the centre leads to no practical error whatever. Again we find, at p. 7, another error is pointed out by Sir Astley; but, as Mr. Earle's words incline us to suppose that he is speaking of what may be produced in the skeleton, and which Sir Astley does not deny, we may be allowed to pass on to the subject of fractures of the neck of the thigh-bone. After some preliminary remarks, our author says:

"The neck of the thigh-bone may be broken at different parts of its extent, either at the junction of the head with the neck, or at the most contracted part, about mid-distance between the head and trochanters, where the parietes of the bone are thinnest. The most frequent situation is at the junction of the neck with the shaft of the bone, in which case the fracture often extends from the root of the trochanter major to the trochanter minor, and is partially, if not entirely, exterior to the capsular ligament. This latter accident is occasionally complicated with fracture of the trochanter major, in one or more places; and in this case the neck of the bone is generally forced between the trochanter and shaft of the bone. It has been remarked that fractures of the neck, at the narrow part, are generally in a transverse direction; a circumstance which may arise from the thinness of the parietes of the bone, which is principally composed of cancellous structure, covered by a very thin layer of compact bony matter.

"The neck of the thigh bone is so protected by the surrounding soft parts and the trochanter major, that it is completely defended from the immediate action of external violence; and, consequently, from any direct fracture. It follows that it must be broken by a contre-coup, either by a fall on the trochanter, the feet, or knees. The former of these is by far the most frequent cause; although, on a superficial view, it may not appear the most probable. Desault says that, in his practice, twenty-four cases in thirty were produced in this way. The experience of Sabatier and Richerand proves the same fact." (p. 19, 20.)

To this rule our author has only met with three exceptions: in two of these (young persons) the apophysis was detached from the epiphysis in consequence of a perpendicular fall on the foot; and, in the other case, the neck of the bone gave way in the capsule, from a mere muscular effort in emptying a pail of water, twisting the body and pelvis at the same moment, while the lower extremities remained fixed.

We find nothing that need detain us in the description of the mode in which these fractures are produced, and come at once to their diagnostic signs. The first point which our author insists upon here is "a remarkable consciousness of incapacity in the injured member, in a person previously in full possession of the locomotive powers of his limb, and when, from the position and direction of it, it is obvious that there is no dislocation." In such a case, he says that a surgeon is fully warranted in treating the case as a fracture, without subjecting his patient to any painful examinations to gratify his own curiosity; a point which he more forcibly urges and insists upon in the following pages, (23 and 24.) This unfortunate paragraph has induced Sir Astley Cooper to express himself in terms sufficiently severe, and to impute to Mr. Earle the heavy charge of teaching incorrect surgical principles. It is, perhaps, to be lamented that Mr. Earle expressed himself in terms so strong and unequivocal upon this point, because it must be obvious to him, upon reflection, that this compendious method of deciding upon an accident of so serious a nature, and which implies a strict confinement of so many weeks, could not be satisfactory either to the surgeon or to the patient. That, in all cases, such an examination as shall convince the practitioner of the correctness of the opinion he is about to give, is necessary, both for the reputation of himself and the welfare of the sufferer; and every practitioner must recollect numerous instances where a violent blow or a severe strain have as effectually deprived a man of the use of the limb for a limited period, as the most complete fracture could have done. In order to get rid at once of a very painful part of our subject, we will in this place remark that, at page 34, Mr. Earle has expressed himself incautiously with regard to the mode of examination recommended by Sir Astley Cooper for the detection of these fractures; but, if that gentleman has erred on the side of prudence, Sir Astley cannot be said to have shown very good taste in the mode of his reply; for he has selected a case of fracture of the neck of the thigh-bone, occurring in a man of the name of Daniel Spilling, a patient at St. Bartholomew's Hospital, in which it appears the limb was examined with great freedom; that, in consequence of the very deranged state of the man's health, but little attention was paid to his limb; and that he died on the eleventh day after his admission. All the points of this case are printed in italics in Sir Astley's "Appendix;" and then he adds, that he would not for the world, if he could, say any thing which would lessen the character, or hurt the feelings, of any of the medical officers of that establishment! Now, if there *had been* any thing improper in the treatment of the above patient, it is by no means clear that the free examinations were made by Mr. Earle, or with his concurrence; and the state of the man's health appeared to render any local treatment of the limb useless. It is to be recollected, also, that the case is merely brought forward by Mr. Earle to prove the small degree of shortening of the limb which sometimes occurs in these accidents.

We are glad, however, to dismiss this part of the subject, and revert to the diagnostic marks. We have already expressed our conviction that, with regard to the first of his arguments, Mr. Earle is in error, and that such an examination as is necessary to establish the nature of the accident is imperiously called for, but no more: nor can we conceive that, when this is done with proper caution and tenderness, there can be any fear of tearing the "reflected layers of the synovial and fibrous membranes," which have escaped the shock of the accident, and upon the integrity of which so much appears to depend.

The next point to be considered is the degree of retraction of the limb consequent upon this injury, and which is one of the strongest-marked diagnostic signs. Our author here controverts Sir Astley Cooper's statement, that the limb is from one to two inches shorter than the other, and (which, by the bye, that gentleman now declares to be much within the truth,) quotes the two following passages from his work on Fractures and Dislocations:* "He (Sir A.) accounts for this retraction in consequence of the trochanter being drawn up as high as the ligament will permit. Three or four hours must elapse† before this appearance assumes its most decisive character, as the muscles require some time to acquire a fixed contraction; and this is the reason that the accident has been mistaken for dislocation." In putting these two quotations side by side, and then reasoning from their apparent contradiction, we cannot help thinking that Mr. Earle has misconceived Sir Astley Cooper's meaning. On an attentive perusal of the first three pages of that gentleman's work, wherein this subject is discussed, it appears to us that all Sir A. means to assert is, that the limb is *generally* shortened from one to two or two and a half inches; that the muscles are the agents in this shortening; that the limb may, however, be *repeatedly drawn down* to the same length with the other; that the term three or four hours applies only to the *degree of eversion of the foot*, and not to the period at which the limb becomes *permanently* shortened; and that when he says "the evidence of the nature of the accident continues until the muscles acquire a fixed contraction, enabling them to resist any extension but what is of the most powerful kind,"

* P. 117. † P. 119.

he by no means intends to limit this to three or four hours; which latter clause, as we before said, appears to us solely to allude to the eversion of the foot, and, consequently, to the mistaking the accident for a dislocation. If this explanation be just, there is then at once an end to this part of the discussion; and the minor disagreements as to the greater or less retraction of the limb will be easily adjustable, since that will depend upon the more direct or transverse nature of the fracture,—on the degree of violence done to the capsular ligament,—and, perhaps, also, in some degree, upon the irritability of the muscular fibre of the person in whom the accident has occurred. Both appear to be of opinion, that, in fractures external to the capsule, the limb is not so much shortened as in the internal fracture.

We shall pass over Mr. Earle's observations on the preparations in the different museums in the metropolis, as far as relates to the degree of retraction, because it is not in dried preparations that this point can be decided. That gentleman has already most handsomely and candidly admitted his mistake with reference to a preparation in Mr. Langstaff's possession.*

With regard to the eversion of the foot as another diagnostic mark, we find our author disposed to agree with Sir Astley, although he inclines to the opinion of Dessault and the French surgeons, that it is occasionally inverted; and one or two such rare instances are mentioned by Sir Astley. With every respect for the memory of the great surgeons which France has produced, we may be allowed to say that, upon this subject, we are not disposed to defer to their authority; because they have confessedly not attended to the distinction between fractures external to the capsule and those that occur within it; as a reference to Mr. Cross's work will sufficiently prove, as well as the preparations which they preserve as specimens of union in the latter instance.

We now come to the period of life at which this accident usually occurs, and which has given occasion to another criticism of Mr. Earle's, originating, if we mistake not, in a misconception of Sir A. Cooper's meaning; and this we can the more readily believe, as we fell into the same mistake in reading the first edition of his work, and at the time took occasion to remark upon the discrepancy which appeared in the statement of that gentleman. In truth, this portion of his book is obscure and loosely expressed; but the explanation in the Appendix, we think, sufficiently clears up the meaning, which is simply that, as fractures external to the capsule usually occur under fifty years of age; therefore, if a surgeon be called to a patient

---

* *London Med. and Phys. Journal* for September.

under that age, he will *generally* be correct in concluding that it is a fracture just external to the ligament, or through the trochanter major; but, as in advanced age *both fractures* occur, no conclusion can be drawn but by a very careful examination. The three cases of external fracture, therefore, are given as illustrating this fact, though they are so unfortunately placed, both in the first and second editions of his large work, as really to appear contradictory of the passage immediately preceding them. Upon these three cases Mr. Earle observes—

"And this is all the evidence adduced to prove that fractures external to the capsule occur in early life,—that they are the result of great violence,—that they are not shortened,—and that they are incapable of ossific union; and upon this testimony the profession are called upon to admit Sir Astley Cooper's easy and decisive solution of all the difficulties which have involved this question: a solution brought forward to explain the errors of many deservedly esteemed practitioners." (p. 50.)

This is hardly fair; because the evidence of ossific union in fractures external to the capsule is to be found in many preparations noticed by Sir Astley Cooper, as well as those related by Mr. Cross, Mr. Colles, and others; and does not, either wholly or in part, depend upon these three cases.

Next in point of order we come to the following question of Mr. Earle's:

"Is there any thing in the structure of the neck of the thigh-bone within the capsule, which renders it unsusceptible of fracture at an early period of life? The answer is, that experience proves that it may be broken at any period, provided the force be so directed as to bear particularly upon this part. Sir Astley Cooper has indeed stated, in common with Ruysch and some other authors, that the same violence which produces dislocation in an adult occasions fracture in old age.

"This explanation appears very specious; but can it be borne out by facts or arguments? Does the same force which produces fracture ever cause a dislocation? I firmly believe not; for it is applied in such a direction as to have no tendency to displace the head of the bone. It is rather extraordinary that, in the very next page, Sir Astley Cooper should dwell at some length on the very slight causes which may produce a fracture, and particularly he mentions trifling falls on the trochanter. I would only appeal to the candour of that gentleman to state whether he ever, in the whole course of his extensive experience, knew of so slight a cause, and a force so directed, productive of a dislocation of the thigh in a young, or indeed in any, person? I would willingly consent to let the issue of the present question be decided by his answer. (p. 53, 54.)

To this question Sir Astley replies by referring to a case of dislocation upon the pubes, produced by a person walking across a paved yard, where one of the flag-stones had been taken up; and he has also known a dislocation upwards produced by the foot being caught in the doubling of a carpet, whilst the person was walking across the room. To these examples we might be permitted to add, that the preparations preserved of fractures within the capsule, both in London, Dublin, and Paris, clearly establish that these are the accidents of old age; for, if they commonly occurred in youth, instances to that effect could not fail of being met with, although, for obvious reasons, they might not be so frequent.

We come now to the prognosis of this accident, and which, indeed, contains the very essence of the point in dispute. We are informed by our author that, by some surgeons, more or less lameness is considered as the unavoidable consequence; and some very great names, from Hildanus to John Bell, are ranged on this side of the question: others think that these cases do not differ from common fracture in any material degree, excepting in the difficulty of treating them. The French surgeons are the principal supporters of this opinion. In order to reconcile these opposites, it will be right, says Mr. Earle, to examine in what respect this fracture does really differ from any other, and the reasons that have been advanced in support of the opinion that union by bone will not take place in this as in any other fracture. (p. 61.) "There can be no question (he goes on to say,) that a material difference does exist between a fracture occurring in the neck and in the shaft of the thigh-bone. When the centre of a long bone is broken, both portions are liberally supplied with blood, as well from the nutrient arteries as from the vessels of the periosteum: but in the present case, when the reflected layers of the fibrous and synovial membranes are torn through, one part is almost deprived of its supply of blood: it can receive none from the smooth articular surface, as that would impede the natural functions of the joint; it must then depend for its nourishment on the vessels it receives through the ligamentum teres, and the synovial membrane reflected over it. This supply, it must be granted, is small, and particularly so in old people, to whom this accident most frequently occurs. To this cause may fairly be referred the length of time required for union; and in some instances, in very old and feeble persons, the quantity may be inadequate to the performance of a function, which requires considerable vigour." (p. 62.)

This leads to some observations upon the experiments of JOHN HUNTER and the Taliacotian operations; but which, we must confess, we do not think bear at all upon the matter in question; and the real reason of the non-union appears to have been clearly pointed out by Mr. JOHN BELL, in a passage quoted by Mr. Earle, and which is too important to be omitted here.

"When the neck of the thigh-bone is fractured, the capsule sometimes remains entire. The capsule is of an insensible nature, entering very slowly into action; and within that ligamentous and insensible capsule is included the whole length of the neck of the thigh-bone. The neck is surrounded with mucous fringes, and the cavity in which it lies is lubricated. The periosteum and ligaments are slow in entering into action, or inflaming even when they are lacerated; but, when they remain entire, *they exclude all connexion of the fractured bone with the muscular parts. Thus, unassisted by any of the usual adhesions, the neck of the thigh-bone is left to its own intrinsic powers;* naked bone is opposed to naked bone, and not very regularly opposed, for the ends of the fractured cervix are so obliquely placed with regard to each other, that more than the usual callus would be required for their re-union; and yet they are so entirely deprived of any support from the surrounding parts, that less callus is produced, often none, they frequently remain disunited. That the part is bloodless, that the surrounding parts are little able to contribute their share in the cure, is the very truth." (p. 67, 68.)

It is true that this passage is contrasted by our author with another in the same work of Mr. Bell, and which is in contradiction to it; but of two opposites one must be true, and we do not see any reason, because we reject one explanation, that we may not be allowed to admit the truth of the other.

We now arrive, however, at Sir Astley Cooper's reasons for believing that ossific union does not take place in this fracture; and the first is, that if the broken extremities of bone in any part of the body be kept much asunder, ossific union is prevented. Now this is given by Sir A. as the first and weakest reason he has adduced: it seems, however, to be borne out by many interesting cases of fracture of the tibia especially; but it is perhaps not necessary to insist upon this, because we have already shown that the neck of the thigh-bone is under very different circumstances to any other bone in the body, and therefore that the more perfect apposition of the parts, which Mr. Earle is of opinion may, by proper means, be effected, would, generally speaking, be of no avail.

The second reason given by Sir Astley, is the want of pressure of one bone upon the other, even where the length of the limb is preserved; and which he thinks, in those cases where the capsule is not torn, would be the consequence of the increased secretion of synovia poured out into the joint, and which, by distending the ligament, prevents the contact of the bones. After a time, however, this fluid becomes absorbed, but not until the inflammatory process has ceased, and ligamentous matter has been effused into the joint. It may, perhaps, be doubted whether this secretion can take place to the extent mentioned by Sir A. Cooper; and it is not quite clear to us that, if it were, the consequence would be that of preventing the contact of the bone. We should be inclined to think that, as the pressure of fluids is equal in every direction, if the joint were distended, it would, as Mr. Earle's experiment seems to prove, have the effect of keeping the bones more in apposition ; but it is, probably, in the majority of instances, not influential either way : and therefore we may pass on to the third and principal reason given by Sir Astley, which is the want of ossific action in the head of the bone when separated from the neck, its life being supported solely by the ligamentum teres. However, he admits that there may be some exceptions, where the bone might be broken without the strong sheath of the periosteum, and reflected ligament which surrounds it, being torn ; and under such circumstances, if they were ever to happen, ossific union might proceed: but then the usual shortening of the limb would not occur. It is this circumstance which Mr. Earle believes to happen in a large majority of instances, and which, consequently, has induced him to come to the conclusion that union is possible, and that the great, if not the sole, difficulty lies in the great mobility of the upper part of the bone, and the little attention which has hitherto been paid to restrain this free motion. Here we must observe, that the experiment detailed by Mr. Brodie, in the Appendix of Sir Astley Cooper, would appear very much to corroborate that gentleman's views ; and it is so much in point that we cannot refrain from inserting it.

"The circumstances of the experiment which I mentioned were briefly these;—The tibia of a guinea-pig was broken at the lower end; a month afterwards the animal was killed. On dissection, I found a fracture extending across the tibia transversely, and so close to the ankle-joint, that it was situated at that part of the bone which is covered by the reflected layer of the synovial membrane. The synovial membrane itself, and the ligaments of the joint, appeared to have been very little injured, and the broken surfaces had remained in good apposition : nevertheless, there was not the smallest union of them, either by bone or ligament, and there had been no formation of callus round the fracture. The bone in the neighbourhood of the fracture had become compact and hard, in consequence of the ossification of the medullary membrane lining the cancelli." (*Appendix*, p. 30, 31.)

Now this experiment tends very much to strengthen John Bell's opinion, and would seem to render it even doubtful whether ossific union could, under any circumstances, take place, where a fracture occurs in that part of a bone covered by the reflected layer of the synovial membrane, even if it should remain untorn.

We have already adverted to the remarks which Mr. Earle has made upon the mode of examining these fractures recommended by Sir Astley Cooper; and have to observe, that Mr. Earle next alludes to the prejudice existing in Sir Astley's mind upon this subject for so long a period, and which (says Mr. E.)

may fairly be supposed to have prevented him from making any very effectual attempts to remove the difficulties attending the treatment. (p. 85.) But what does Sir A. Cooper say in reply to this? He says, that in early life he made use of "all appliances, and means to boot," to effect the union of this fracture; and that it is a firm conviction that the plan he at present adopts is the easiest, and finally restores to the patient an useful limb, by crutches,—by a high-heeled shoe,—and by the aid of a stick, until he can walk without one, that alone has induced him to adopt and recommend it: adding, that he has witnessed the practice of Mr. Cline, Mr. Chandler, and many others, all of whom tried various means for uniting a fracture of the cervix femoris within the capsule; and all of them unsuccessfully. (*Appendix*, p. 33.) Nay, he goes further; for he contends that this non-union is a wise provision of nature, applicable to the patella, olecranon, and the extremities of bones within the articulations: "for I find (he says,) that, if the neck of the thigh-bone be shortened, and falls towards the shaft of the bone, the person is unable to move his hip-joint, and becomes what is vulgarly called bed-ridden. The cause of this is easily explained; for, when the cervix is shortened, the trochanter falls upon the acetabulum, and the play of the thigh-bones become destroyed. In an ossific union of the cervix femoris, if such should ever occur, as the neck of the thigh-bone is greatly shortened very soon after the accident, the same effect would be produced, and the play of the limb be destroyed; more especially when the callus, by which the union would be produced, must occupy a considerable part of the acetabulum, and the patient's state would probably be much worse than in that of ligamentous union." (*Appendix*, p. 32.)

Mr. Earle devotes three pages to the consideration of Sir Astley Cooper's experiments on animals; but we must be allowed to say, that it does not appear to us that his objections materially affect the deductions that Sir Astley has formed from them. In fact, from that gentleman's reply, it would seem that the objection to the experiments, on the score of the fracture being compound, did not exist at all in one of the instances; and that, in fact, it does not apply to any of them, as the wounds were healed by the first intention; neither was the capsule opened in all the experiments. The only suggestion of Mr. Earle's, which appears to us to have any weight on this point, is that no care was taken to prevent the motion of the part; at least, no precautions are mentioned: neither does Sir Astley, in his reply, explain that circumstance in a satisfactory manner; for, although the muscles would naturally draw up the limb, and the animal would instinctively avoid bearing upon it, that is a very different thing from preventing all motion by amputation, as Mr. Earle recommends. Our author, however, appears aware that it will be expected of him to do more than reason on the side of the question which he has adopted; and we certainly think that the *onus probandi* does rest with him to show that bony union has taken place in these fractures. We are compelled to say that Mr. Earle's proofs are the least satisfactory part of his performance: they amount, indeed, but to the solitary case of Mr. Stanley's, and to which, as Sir Astley himself seems disposed to attach some importance, we give entire. These specimens were found in the body of a subject in the dissecting-room.

"As it is Mr. Stanley's intention to publish a description of these bones, I will only so far anticipate that gentleman's account by stating that they were both found in the same subject; that the fracture on the right side was entirely within the articulation, and on the left side partially; that there was very little shortening of the limbs, arising only from the loss of obliquity in the neck; and, lastly, that the most perfect osseous union has taken place, which can be traced through the whole substance of the neck, in the different sections which Mr. Stanley has made.

"This case must, I think, be admitted by the most sceptical, and must at once place the possibility of such an occurrence on the firm basis of actual demonstration. Nothing is known respecting the case, either as to the mode of treatment, or whether both the bones were fractured at the same time." (p. 99, 100.)

The case of the nurse belonging to St. Bartholomew's Hospital is certainly a failure; and that from the Journal of Erndleus cannot detain us for a moment. It is remarkable, indeed, that no other proofs than these three could be found to controvert an opinion which rests upon the evidence of so many preparations in so many different collections, and which is further strengthened by the circumstance of the examination of those in Paris by Mr. Cross; from whose accounts it appears that there were only three that could for a moment give a colour to the opinion of ossific union taking place within the capsule; and yet not one of these three preparations would stand the test of a rigid scrutiny. Let it be recollected that, of the forty-three instances adduced by Sir Astley Cooper, only twelve are from St. Thomas's, six are to be found in the collection at St. Bartholomew's; and yet the dissecting-rooms of London have not, during the whole time that this question has been agitated, been able to furnish, exclusive of Mr. Stanley's, a single unequivocal specimen on the contrary side of the question. This is very strong, because, although we willingly admit the ingenuity with which Mr. Earle has supported his opinion, and the skill which he has displayed throughout his argument, we must not suffer ourselves to yield our conviction against the evidence of facts, which so strongly corroborate Sir Astley's views. Let it also be recollected that this latter gentleman has distinctly admitted that there are two circumstances under which these fractures might possibly unite, but they are so rare that he has never met with them; and therefore, he observes, that they cannot shake the truth of a *general* principle.

With regard to the case of Mr. Stanley's, we think it but just to give Sir Astley's remarks upon it, and then leave our readers to draw their own conclusions:

"The description which our author has given of this case is defective in two very essential points. In the first place, he does not say that in one of them the appearance of fracture was external to the capsular ligament, and, therefore, not applicable to his object. Secondly, he does not mention any thing of disease which existed in other joints.

"I do not mean to deny the possibility of the necks of both thigh-bones in this subject having been fractured, because that point can only be determined by the history of the accident, and by a very careful and accurate examination of several sections of the bones; but I can show that similar effects are produced by disease.

"The neck of the thigh-bone, in adult persons of middle age, has a close cancellated structure, with considerable thickness of the shell which covers it; but, in old subjects, the cancellated structure of the shaft of the bone, which is formed of coarse net-work, loaded with adipose matter, is often extended into the neck of the bone, and the shell which covers it becomes so thin that, when a section is made through the middle of the head and cervix, it is found diaphanous: of this I have several specimens. As the shell becomes thin, ossific matter is deposited on the upper side of the cervix, opposite the edge of the acetabulum, and often a similar portion at its lower part, and thus the strength of the bone is in some degree preserved: this state may be frequently seen in very old persons. Mr. Steel, of Berkhampstead, one of the most intelligent surgeons and most respectable men I know, gave me the thigh-bone of a person thus altered, whose age was ninety-three. When the absorption of the neck proceeds faster than the deposit on its surface, the bone breaks from the slightest causes; and this deposit wears so much the appearance of an united fracture, that it might easily be mistaken for it." (p. 38, 39.)

That so few instances of success in the treatment of these accidents should be found, Mr. Earle explains by the treatment which Sir Astley Cooper has adopted himself and recommended to others, and which treatment renders it next to a miracle (we use Mr. Earle's words,) that ossific union should take place: but it must be recollected that Sir Astley distinctly says, that his present treatment is only the result of many trials of other modes, which have all failed, both in his hands and those of some of the first surgeons of this town; and that he still is of opinion that it should not be resorted to whenever there is any reasonable doubt as to the nature and extent of the fracture, and consequently, according to his belief, any chance of a more perfect consolidation of the parts.

We need not detain our readers with any description of the mode of treatment recommended by Sir Astley in these cases, since his work is so universally known, but draw this long article to a conclusion, by describing the mode of treatment as detailed by Mr. Earle: and here we have much pleasure in being able to say that, whatever opinion may be entertained as to the main question we have endeavoured to discuss, that this portion of Mr. E.'s book will be read with pleasure and profit by every surgeon, the subject being one of high importance and much difficulty. Mr. Earle has given a concise, but clear, idea of the different positions and plans that have been recommended for the cure of fractures, both of the shaft and neck of the thigh-bone, including Dessault's, with Boyer's improvement, and Mr. Hagedorn's more recent contrivance, and then adds—

"Before concluding my observations on the straight or extended position, it will be right to mention one case, in which every straight splint, even that proposed by Hagedorn, would be perfectly unavailing,—namely, when the fracture is just below the trochanter minor; in which case the psoas magnus and iliacus internus muscles often draw up the upper portion so forcibly towards the groin, as to cause a very evident projection at that part; and, if the bone should unite in this position, the most distressing lameness and deformity must be the inevitable consequence.

"In this case, which forms an exception to the general rule of the lower portion of the fracture causing the deformity, it is necessary to bend the thigh very much on the pelvis, almost to a sitting posture, to facilitate the approximation of the broken ends of the bone. Any attempt to employ the extended position, under any modification, would be nearly certain of failure, unless the spasmodic action of the psoas and iliacus could be effectually overcome, and the bone replaced in its situation ; which would be very difficult to accomplish, in consequence of the shortness of the lever and the want of antagonist muscles. This case, however, is certainly of rare occurrence, and the treatment of it is attended with peculiar difficulties. (p. 114, 115.)

Mr. Earle next proceeds to examine the plan of treating fractures of the thigh recommended by Sir A. Cooper, in fractures of the shaft of the bone, and in those of its neck external to the capsule: the principle is that of the double inclined plane, first suggested by Mr. White, of Manchester, and improved by Mr. James, of Hoddesdon. To this principle our author is friendly, but he suggests some imperfections in that described by Sir Astley, and then proceeds to describe his own labours in this department. We much regret that we cannot do justice to the very ingenious contrivance of Mr. Earle: the description of the bed which he invented is in itself necessarily long, as there is some complexity in its parts, and, without the assistance of the engraving, we despair of being able to make ourselves understood. The apparatus he at present employs is, however, in many respects different from that which first suggested itself to his mind, and which was rewarded by the Society of Arts and Sciences, twelve years ago. It consists of a modification of the double inclined plane.

"The bed on which the patient is placed is divided into three portions,—the upper one for the trunk, the short middle one for the thighs, and the lower division for the legs. These admit of being placed at various angles and in different positions. The following are the advantages gained by this apparatus:—When the patient is placed on the bed, the pelvis will, from its own gravity, remain fixed at the bottom of the angle formed by the superior and central inclined planes; and the aperture made in the central part readily admits of the patient relieving himself, and being properly cleansed, without the least movement of either trunk or extremities. Should it be desirable, in young persons, or under particular circumstances, to secure the pelvis more firmly, it may be easily accomplished by two broad straps, brought from the edge of the aperture, and passed obliquely round the upper and outer part of the thighs, which should pass once round the pelvis, and be attached to buckles at the outer side of the mattress. By this simple plan the possibility of motion of the pelvis is prevented, and firm compression may be applied over the trochanter. This will, however, very rarely be required, as, generally speaking, the weight of the pelvis is sufficient to keep it steady; and no other bandage is requisite than that which secures the feet to the foot-boards. The position of the patient, namely, on the back, on a gently-inclined plane, with the thighs and legs half bent, and the whole equally and firmly supported on a level surface, is one peculiarly easy and comfortable, and can be longest endured without complaint. The knee being bent over a double inclined plane, affords the best and easiest means of making permanent extension, by placing the fulcrum under the ham, and making a lever of the leg, whilst the foot is securely fixed to the foot-board, and all eversion or inversion prevented. The gradual curve formed by the mattress on the double inclined plane, is exactly adapted to the naturally arched form of the thigh-bone, and is the least likely to cause any derangement in the length and direction of the broken limb. The central division of the bed admitting of being drawn out to the extent of several inches, enables the surgeon to adapt it to the exact length of the thighs of different individuals. The juxtaposition of the limbs affords constant opportunity of minutely comparing them, and of observing whether they exactly correspond. The apparatus for fixing the feet at the same time supports the bed-clothes, takes off pressure from the heels, and maintains the limb at its proper length. By fixing both feet to the foot-boards, all motion of the pelvis and lower extremities is more effectually prevented; for, when the sound limb is left at liberty, the patient is very apt to move it, and to shift his position from the central aperture." (p. 126 —128.)

We must now take our leave of Mr. Earle. The remaining part of his work we cannot at present touch upon, as we have already devoted so much space to the consideration of one only of the subjects contained in it. We lament that we have been obliged conscientiously to give an opinion in opposition to that gentleman, for whose talents and attainments we entertain the highest respect; but we have a public duty to perform, and we must not shrink from the performance: our motto must be—"*Amicus Plato sed magis amica veritas.*"

We have, in the foregoing pages, necessarily anticipated almost all we had to say relative to Sir Astley Cooper's *Appendix*. We might find fault with its composition,—we might suggest that in some passages there is a tone of severity and harshness, which a man so eminent need not have employed; but we have promised to avoid all remarks that might tend to keep alive or increase irritation, and therefore, lest we should be tempted to break that promise, we shall thus abruptly conclude.

---

### DIVISION II.

### FOREIGN.

ART. IV.—*Memoir on some recent Discoveries relative to the Functions of the Nervous System: read at the Academy of Sciences, 2d June*, 1823. By M. MAGENDIE, &c. &c.—pp. 25. Paris: Mequignon-Marvis. 1823.

BEFORE we make any comment on this Memoir, we shall lay it verbatim before our readers.

At a period not very far distant from us, physiology was but a strange medley of ingenious fictions and subtleties always obscure; a crowd of chimerical beings, spirits, humours, and principles, received a real existence; and what was called a science was, in fact, a sort of fable or poem, in which these imaginary beings were the principal personages; and the action, and the different parts, varied according to the will of the authors, or according to the taste of the age or the spirit of the schools: in short, physiologists were poets and romance-writers, but never observers, and living nature remained unknown. The seventeenth century, that memorable epoch in the history of the human intellect, gave birth to a physiology of a different kind. This physiology neither invents nor creates: it studies and observes; precise experiments, accurate facts, and rigid deductions, are alone admitted; and, if it does not yet possess all the exactness of the physical sciences, that must not be attributed to the method of study, but solely to the nature of the phenomena of which it treats. The Academy of Sciences has always received with indulgence, and seconded by all its means, this physiology, which searches only for the truth; and it is, without doubt, for the purpose of giving its labours a quicker and a surer march, that it now accepts the gifts of that respectable man, who, at his death, made so noble and useful a disposition of his fortune. It is, then, to be hoped that the mysteries of life, of which man has been so long the witness without comprehending them, will be successfully unveiled; and that he will be able to make the same conquests in the living world, that he has been able to achieve over inanimate nature. This hope is at present almost realised. In all the civilised parts of the globe, a generation of physiologists is rising up, who, disdaining systems, spread over living nature the light of the physical sciences, submit them to the proof of experiments, and thus are enabled to discover some of those phenomena which have been concealed until now.

I will not attempt to retrace collectively all the discoveries which have emanated from this happy concurrence of efforts; the territory of real physiology is too much extended to render it possible to run over it in a moment: it is sufficient for me to explain how, in one particular of the most important part of the human organisation, our new lights have been acquired; and to show, by one example, what is the influence of the method by experiment upon the whole science. The point upon which I dwell is especially worthy of regard: it relates to the Brain and Nerves,—to that white or grey pulp which sometimes, united in a mass more or less considerable, fills the head and the cavity of the spine, and sometimes, under the form of filaments of extreme tenuity, establishes a direct communication between all the external and internal parts of our bodies and the central pulpy masses.

Such is the Nervous System. Its functions, connected entirely with our moral, and in a great measure with our physical existence, have been an object of continual meditation to the philosopher, moralist, and physician; and, what is remarkable, the object which appears the most difficult to attain in this double study, the determination of our moral faculties, is precisely that which has been obtained with most speed and certainty. It is sufficient to mention upon this point the immortal works of Locke and Condillac, and the more recent labours of Dugald Stewart and M. De Tracy. We are far from possessing notions equally extended and precise on the physical faculties of the nervous system, in spite of the labours of Halle and his school, those also of Bichat and Legallois: we are in possession only of a small number of important facts upon a question which interests us on so many accounts.

It has long been known that the nerves give sensibility to our organs, and movement to our muscles; that the brain appeared more particularly destined to the phenomena of the intellect, the cerebellum to motion; but what was unknown for a much longer time, and which the beautiful experiments of Lorry and Legallois have placed beyond all doubt, is that the spinal marrow is the most useful portion of the nervous system. There, is to be found the principal seat of sensibility, and the source of all our movements; there, resides the imperious instinct which causes us to respire; so that, strictly speaking, it would be possible to live without the brain or cerebellum, but life, without the spinal marrow, cannot possibly be maintained a single moment. Here, then, are some new facts, which recent discoveries have enabled us to add to those important ones, but which as yet have been but scarce.

One of the most curious discoveries is due to an English physiologist:

it has reference to that admirable faculty by which our countenance becomes the faithful image of the sentiments which agitate us. It was not doubted that the muscles were the agents of the expression of the countenance, and that the nerves directed their several contractions. But the face receives many distinct nerves, and particularly two on each side,—one of which is called the *facial nerve,* the other the *maxillary nerve.* Mr. C. Bell, who has paid great attention to the nervous system, and who has written a "Treatise on the Expression of the Countenance in Man and Animals," questioned which of the two, the facial or the maxillary nerve, was the agent of communication between the muscles of the face and the internal sensations.

To judge of this, it was necessary to make an experiment, which consisted in cutting one of these nerves, and leaving the other entire. The experiment was made upon an ass. This animal was, perhaps, not the most proper to choose, in order to judge of the physiognomy: however, as its passions are sufficiently vivid, it cannot be said to be devoid of expression. The facial nerve of an ass was therefore divided, and it was immediately seen that all motion had ceased on that side of the face where the division had been performed, particularly those of the eyelids and lips. Food was offered to it: on the side where the nerve was undivided, its appetite was vividly expressed; the opposite side remained dead and inexpressive. This was not the case when it laid hold of its food: the same parts which were immoveable as far as regards the expression of the countenance, performed their proper functions in mastication.—Another experiment was made: it consisted in cutting the maxillary nerve, leaving the facial nerve entire. This was performed on another animal; and it was found that the movements of expression had not lost any portion of their activity, whilst those which had a reference to the performance of mastication had ceased entirely. In this experiment, an important remark was also made: the animal had entirely lost the sensibility of that side of the face in which the nerve had been cut, although one of the two nerves distributed to this part remained entire. This experiment deserves to be repeated on some animal whose features have a more marked expression than those of the ass.

A monkey, of the most expressive countenance that could be found at Exeter 'Change, was therefore chosen, and the facial nerve on one side was divided: he lost entirely on that side the power of grinning, *(grimacer,)* and the whole physiognomy assumed, in consequence of the contrast of the two sides, so singular an expression, that it was impossible, on beholding it, to refrain from laughter. All of the assistants in this experiment were struck with the analogy that existed between the countenance of this monkey and that of a celebrated English mimic. It appears very probable that this man was indebted to a natural infirmity for his powers of diversion, and the conjecture was thus verified.

We have repeated the above experiments, and find them perfectly exact. They throw a great light upon the functions of the nerves of the face; they prove, in an incontestible manner, that the motions of the eyelids, nostrils, lips, &c. which form the principal play of the countenance, are depending upon a particular nerve; and that the sensibility of these parts, and the motions relative to mastication, are also depending upon an especial nerve.

These results are not only curious as far as they relate to science, they have also an immediate application to the cure of disease. The countenance is often the seat of affections which bear particularly upon the organs of expression; the mouth is twisted, the eyelids are paralysed, &c. Do not the means of cure become more easy and more sure when the mechanism of the diseased organs is better known? It is thus that the discoveries of physiology will become sooner or later the means of perfecting the science of medicine. Feeling and motion are the two phenomena upon which the actions of external life turn. In a state of health these two phenomena are so united with each other, that they appear to form only one; but, in disease, the separation is sometimes so decidedly marked, that a part of the body, or even the whole of it, loses its sensibility entirely, without in the smallest degree losing the power of motion; and, under other circumstances, it loses its motion, whilst it preserves its sensibility.

These facts, known since the origin of these diseases, have been the objects of the research of physicians of every age; it has been concluded, and with reason, that there must be, in the nervous system, nerves of sensation and nerves of motion. But neither the most minute anatomy, nor morbid appearances after death, nor experiments on living animals, had been able to distinguish nerves of sensation from those of motion. I have lately been led to establish this distinction; and that which hitherto had appeared an insurmountable difficulty, is found to be one of the most simple phenomena of the nervous system.

To understand this result, it must be recollected that all the nerves of the body and limbs have their origin in the spinal marrow; but the manner in which they proceed from this trunk ought to be carefully remarked. They have two orders of roots: one are attached to the anterior part of the marrow; the others, on the contrary, are affixed to its posterior part. These two orders of roots are separated at first by a pretty considerable interval, but afterwards they are re-united and confounded together, forming only one nerve.

I have proved, by direct experiments, that these distinct roots have also functions entirely distinct; the anterior are destined for motion, the posterior for sensation. If the first are divided, the animal loses the power of motion, and *vice versa:* if the second are cut, sensation is lost, but the animal preserves the power of motion. There is, therefore, no longer any difficulty respecting these two orders of nerves,—one destined to motion, the other to sensation. It may be also understood why the ancient anatomists did not arrive at a knowledge of the distinction; they operated upon the nerves only after the re-union of their roots in a single fasciculus, and it was therefore impossible for them to separate those filaments which are destined for sensation from those that are peculiar only to the performance of contraction.

I have recently had occasion to confirm, in man, the different functions of the roots and of the nerves. A person had lost the power of moving the arms for some years, but he had preserved the perfect sensibility of these parts; he died, and, upon examining the body, the posterior roots were found entire; whilst the anterior roots, evidently changed, had lost their medullary substance, and were reduced to their membranous sheath. The nerves give motion and sensation to our organs only because they are attached to the spinal cord: whenever they are isolated by a wound, or by any other cause, the parts to which they are distributed become immovable and insensible. It was therefore an object of curiosity to ascertain if the spinal marrow itself was not divided into halves,—one destined to motion, the other to sensation. In physiological researches, conjectures, which only rest upon analogy, are often contradicted by experiments: here, on the contrary, experiment fully confirmed conjecture.

I observed that the spinal marrow is formed, as it were, of two cords joined together; one of which is endowed with exquisite sensibility, whilst the other may be said to be a stranger to this property, and appears reserved for motion. I have proved the reality of the separation of the two properties in the whole length of the spinal cord; and, as it is proved, in the beautiful experiments of Legallois, that all the other organs draw their sensibility and motion from this, we are led to this remarkable consequence, that we must search in vain for one single point in the whole body where sensation and motion are confounded together. Accordingly, it becomes extremely probable that, in the case of those persons who lose the power of motion and preserve that of sensation, and reciprocally in those who lose their sensibility and preserve the power of motion, that there is disease either in the sensitive cord or in the motive cord of the spinal marrow.

Accident has determined, for chance has also its influence in the progress of science, that a patient *(aliené)* of the hospital of Charenton had for more than seven years lost the power of motion in the whole body, although the sensibility was preserved; he died last month. M. Royer-Collard, physician of the establishment, examined the spinal cord with the greatest care, and a most marked alteration was found in the whole part of the marrow destined for motion, whilst that part which is the seat of sensibility was perfectly entire. Thus there is no longer any doubt as to these two grand phenomena of our physical life. They have each their distinct organs; and, if it happens that sensation and motion appear to be confounded in one act, that arises probably from the continuity of their organs.

Whilst I was devoting myself to these researches relative to the spinal marrow, I had occasion to make a remark, which appears to me not devoid of interest. It might be believed that the properties of this part are more decided in proportion as we penetrate more profoundly into the tissue of which it is composed, and that its centre is, if we may be allowed the expression, the sanctuary of sensation and motion; but the precise opposite to this is the fact. The centre of the spinal marrow is not sensible, and, on touching it, no movement is excited: it is at the surface of this organ that its properties, both of sensation and motion, are especially developed. Those who think that the electric fluid circulates in our nervous system, may draw from this fact a new argument in favour of their opinion; for electricity is, as we know, seated on the surface of those bodies which it affects. I have no occasion to remark, that the facts I now relate will have a great influence in the treatment of various paralyses. How can one now treat by the same means a palsy of sensation and one of motion? The organs being different, the curative means ought not to be the same. I am happy to say, that already many distinguished physicians, who are unwilling that the science of medicine should continue an uncertain course in the midst of vain hypotheses, have obtained the most marked advantages from this physiological distinction, in the cure of palsies.

It would doubtless be of the highest importance to know how sensation and motion, which have their seat in the spinal cord, as we have already said, are propagated in the head, and extend to the brain and cerebellum; or, in other words, to know how impressions received by the sense, and the determinations of the will, are transmitted to the spinal marrow. Here the difficulty of making experiments becomes extreme; and I must candidly avow that I have arrived at no positive conclusion on this question, which touches upon the most secret principles of life. But the great number of fruitless essays which I have attempted have enabled me to establish a fact, which appears to me worthy of fixing the attention of physiologists, and upon which, as far as I know, nothing has hitherto been said. When an animal is deprived of the cerebral hemispheres, he runs straight forward with a singular rapidity, and as if he were pursued: it might be said that an irresistible force pressed upon and precipitated his motions. If, on the contrary, the action of the cerebellum be arrested, his motions take altogether an opposite direction,—the animal falls backwards; and it is a remarkable phenomenon to see a bird, for instance, in which the cerebellum has been slightly touched, no longer to make any motion whole days together, either in walking, swimming, or flying, but in a direction backwards. It would seem, then, to result from these experiments, that an animal, in its ordinary state of health, is placed between two forces which are in equilibrium, one of which would always impel him forwards, whilst the other would push him backwards; and the will would have the power of disposing, at his pleasure, of these two forces. A disease of the horse, but little known, is proper to verify the exactness of these results. Veterinary surgeons call this disease *immobility;* and, in fact, when one wishes to pull back an animal seized with this disease, he remains immovable, whatever means or force is employed. Forward movements are, on the contrary, easy; and appear sometimes, indeed, to take place without the participation of the will. If the consequence which I am about to deduce is correct, this disease ought to consist in a physical alteration of the brain, or in some interruption to the action of that organ.

In the last month I examined two horses attacked with immobility, and my conjecture was completely verified. In each the brain was visibly altered; the cerebellum, on the contrary, was perfect.

It appears, then, to be demonstrated, that the two opposite moving powers of the brain and cerebellum exist in animals, and that, in certain cases, they can be rendered independent of the influence of the will. Is this the case in man? Our motions, which execute with so much precision the orders of the will, can they cease to be obedient, and fall into a state of anarchy? In fine, the faculty of the will, is it distinct from that which directs our movements? One dares scarcely attempt to remove these doubts; we seem to arrive at those abstractions, eternal boundaries of human reason; and, nevertheless, I have seen, and have been able to study for many weeks, with a man of great knowledge, and fully capable of observing himself, the complete separation of the will and the power which directs our movements. In consequence of a violent chagrin, the person of whom I speak lost suddenly, and greatly to his surprise, the influence of the will over his motions: in spite of himself, he felt compelled to assume the strangest attitudes, and to make the most extraordinary contortions. Language cannot paint the multiplicity, the strangeness, of his motions and of his positions: in some instances, his movements were of the ordinary kind; again, without his will having any share, he was seen to rise and to walk on precipitately, until he came in contact with some solid body which opposed itself to his passage; sometimes he turned back with the same quickness, and was only stopped by the same cause. He often resumed the power of certain movements, without being in the slightest degree able to direct others. Thus the arms and hands frequently obeyed the will, and still more frequently those of the face and of speech. He was frequently able to go backwards at the moment that he found it impossible to advance, and he then employed this retrograde movement to arrive at the objects of which he was in search. This condition lasted upwards of four months, and ended in the most fortunate manner. A few grains of a substance that chemistry has just discovered (the sulphate of quinine,) were sufficient to render all the movements obedient to the will.

One would, then, be induced to think that the faculty of the will is a different thing from the faculty of producing and forming our movements into regular acts. This is the only consequence that I wish to deduce from the fact which I have recorded. Many others will, no doubt, present themselves to our minds; but, in order to pursue them, it will be necessary to become a metaphysician, and I wish to remain a physiologist only. Thus, the object of this Memoir, as we have already announced, is not so much intended to display all that may be said relative to the nervous system, as to show how we have been led to make those discoveries, which have recently been made on a subject so important to know, and so difficult to study! Our task is finished, if, in showing what have been the results of the experimental method in one of the departments of science, we have caused you to feel what ought to be its influence upon the science generally.

May, then, this happy method, the only one which is adapted to the natural sciences, attract towards it all those who feel a sincere interest in the progress of knowledge. May science, according to the fine expression of Bacon, march for a long time with a firm step in the new path which she has just entered, and thus multiply those discoveries which do honour to the intellect of man, and contribute to protect his existence.

The plagiarisms of the French in matters of science have been so frequent and so flagrant, that they cease now to excite our astonishment. There is scarcely a new fact discovered,— scarcely a new scientific law established, in England or Germany, which does not find a claimant among our French neighbours. To a certain degree the public might be deceived by these attempted impositions, but, when multiplied as they have been in France, they only admit of one name and of one interpretation. A late eminent reviewer has so fully exposed their gross deceit, that any detailed proof of these assertions would be unnecessary. But we little expected that M. Magendie, who, by his ingenuity and perseverance in the study of physiology, has merited eminence among the followers of science, should have forced from us such severe censure.

Who, on reading the preceding Memoir, would suppose that any thing further had been done of late years by English physiologists, in regard to the nervous system, than their having made some physiognomical observations on the actions of the muscles of the face, and on the nerves by which they are controlled. Such would be the impression conveyed to one ignorant of what has been done in this country, on reading this Memoir presented to the French Institute. If the members of that learned and *liberal* body act upon this occasion as usual, instead of reprobating, they will applaud and crown the plagiarist.

To Mr. Charles Bell, in this country, is due the honour of having first proved the truth of the great and important law, that different portions of nervous matter bestow distinct functions on the parts of the body to which they are distributed. M. Magendie himself, as will be shown in the sequel, bears witness to the truth of this statement; while he has neither done, nor even attempted to do, more than follow out the general law in some of its petty details; by which, however, it must be admitted, the correctness of Mr. Bell's views has been corroborated.

Mr. Bell, in the first paper delivered to the Royal Society, says, that he does not detail his discoveries in the order they were made, but in that manner which his friends thought best calculated to excite attention; an object in which he has at length succeeded.

He presented his observations on the Nerves of the Face first, because the facts were distinct, easily proved, and of such a nature as at once to arrest attention. He wished to show that nerves, hitherto considered of the same nature, were essentially different, and possessed distinct powers: for this purpose he commenced with his paper on the Portio Dura and on the Fifth Pair.

But the course of his discoveries, as detailed in his public lectures, were in this order. The examination of the anatomy of the nervous system led him to conjecture that any tract of nervous matter, wherever continued, preserved the same function in all its extent; and that the different endowments belonged to distinct fasciculi, or tracts of nervous matter.

In a paper in the 286th Number of this Journal, a short history is given of the progress of his discoveries; and, in his public lectures, Mr. Bell has been in the habit of stating it nearly in the following manner: Looking for the origin of a nerve, purely a motor, he endeavoured to follow the tract of matter from which it sprung, and then to see if all the nerves arising from the same tract were not also motors.

He fixed on the third—a nerve of motion; he traced the nervous cord of matter from which it rose, to the roots of the sixth, a nerve of motion, and also to the roots of the ninth, a motor of the tongue. He then traced the same cord of medullary matter into the spinal marrow; and, seeing that the anterior roots of the spinal nerves were still in the same line, he made experiments to ascertain whether they were not nerves of motion; and he found them to be so.*

Observing, with renewed interest, the origins of the spinal nerves, and having ascertained that it was only the anterior roots which gave motion, he was desirous of discovering the double office of the spinal nerves, and especially of ascertaining whether it could be possible that a ganglion, instead of being, as generally supposed, a body to cut off sensibility, was a mark of the nerve upon which it was formed, being one of sensation. With these views, he prosecuted the inquiry into the functions of the nerves of the head; for, if the fifth (being a ganglionic nerve,) bestowed sensibility, then the objection that the posterior roots of the spinal nerves could not be nerves of sensation, because they had ganglions on their roots, would be removed. He found the fifth pair of the encephalon to be, in all essential circumstances, the same with the spinal nerves, having even a ganglion on one of its roots, similar to those on the nerves of the spine; and, on dividing it, he found that all sensibility in the head and face was destroyed.

This single discovery of the fifth being analogous to the spinal nerves, removed all the obscurity which hung over the subject. Had Mr. Bell been a mere *experimentalist*, he would have fallen into the mistake that the sole office of the fifth was for sensibility; but, having set out with the observation that it was a double nerve, like the spinal nerves, and that, by having

* See the conclusion of these remarks.

two roots, it had two distinct functions to perform, he discovered, further, that it was not only the nerve which bestowed sensibility, but that which performed certain muscular motions common to all classes of animals.*

He had now made out the grand systems of nerves which bestow sensibility, locomotion, and agency, in all classes of animals. He had shown that there was a class of symmetrical or regular nerves, going off from the spinal marrow in succession from the head to the sacrum. He next, in a manner which appears to us satisfactory, explained the reason of certain other nerves being bestowed on the higher classes of animals, for the purpose of combining respiration with certain voluntary actions. He showed, by a most interesting suite of experiments, that the portio dura was the respiratory nerve of the face; that it was through this nerve, that the motions of the cheeks, and lips, and nostrils combined with the motions of respiration in speaking, singing, sucking, drinking, spitting, coughing, and sneezing; that in all these actions there was necessity for a combination of the act of volition with the act of respiration, and therefore that the portio dura had a different origin, and had a different course from the fifth pair.

These observations, in ingenuity and importance, are almost unexampled in the history of physiology; but who, we repeat, could guess that this was the state of our knowledge in this country, from reading the partial and meagre exposition submitted to the French Institute? or who could have believed that M. Magendie had all Mr. Bell's papers and his original Essay on the subject lying by him while he was composing this Precis? But that he really had the Essay, of which the following is an extract, in his possession, is proved by his having made a long quotation from it, in his Journal for October 1822.

"I took this view of the subject: The medulla spinalis has a central division, and also a distinction into anterior and posterior fasciculi, corresponding with the anterior and posterior portions of the brain. Further, we can trace down the crura of the cerebrum into the anterior fasciculus of the spinal marrow, and the crura of the cerebellum into the posterior fasciculus. I thought that here I might have an opportunity of touching the cerebellum, as it were, through the posterior portion of the spinal marrow, and the cerebrum through the anterior portion. To this end I made experiments, which, though they were not conclusive, encouraged me in the view I had taken.

"I found that injury done to the anterior portion of the spinal marrow convulsed the animal more certainly than injury done to the posterior portion; but I found it difficult to make the experiment without injuring both portions.

"Next considering that the spinal nerves have a double root, and

* See the Number of this Journal for October 1822.

being of opinion that the properties of the nerves are derived from their connexions with the parts of the brain, I thought that I had an opportunity of putting my opinion to the test of experiment, and of proving, at the same time, that nerves of different endowments were in the same cord, and held together by the same sheath.

"On laying bare the roots of the spinal nerves, I found that I could cut across the posterior fasciculus of nerves, which took its origin from the posterior portion of the spinal marrow, without convulsing the muscles of the back; but that, on touching the anterior fasciculus with the point of the knife, the muscles of the back were immediately convulsed.

"Such were my reasons for concluding that the cerebrum and cerebellum were parts distinct in function, and that every nerve possessing a double function obtained that by having a double root. I now saw the meaning of the double connexion of the nerves with the spinal marrow; and also the cause of that seeming intricacy in the connexion of nerves through their course, which were not double at their origins."—(*Idea of the Anatomy of the Brain*, by Charles Bell, 1809.)

If the above quotation is not sufficient to prove M. Magendie's knowledge that the experiments on the roots of the spinal nerves had been made even before he began his career as a physiologist, we may add the following, from a communication to the Medico-Chirurgical Society by Mr. Shaw; and which we have reason to believe is also in M. Magendie's possession.

"I shall, however, take the liberty of trespassing still more upon the time of the Society, by making a few remarks upon a very curious question, which has particularly excited the attention of physicians in all ages since the time of Galen:—Why sensation should remain entire in a limb, when all voluntary power over the action of its muscles is lost? or why muscular power should remain when feeling is gone?

"The attention of Galen was particularly directed to this question, in consequence of his having been called upon, by some of his contemporaries, to account for the manner in which he had cured a partial paralysis of the finger by application made to the spine.

"In answer, Galen told them that two sets of nerves went to every part: one, to endow the skin with sensibility, the other to give the muscles the power of voluntary action. This opinion was probably founded on a mere theory; but the facts lately discovered, and the observations which have been noted in attending to the phenomena of disease, though they do not afford absolute proofs of the correctness of Galen's supposition, still they go far to establish the fact, that every part of the body which is endowed with two or more powers is provided with a distinct nerve for each function.

"The form of the nerves which at the same time endow the skin with sensibility and the muscles with the power of voluntary motion, is such, that they appear to be single cords; but, if we examine the origin of any of those nerves, we shall find that it is composed of two packets of fibres, which arise from distinct parts of the spinal marrow. These origins are soon enveloped in the same sheath, so as to appear, to a superficial observer, to form a single nerve.

"It is not too much to suppose that either of these origins may be affected, while the other remains entire. To prove this by ocular demonstration will perhaps be impossible, and therefore the question will probably remain undecided. But we have already seen examples of the consequence of injury to a nerve that has a single root,—viz. the portia dura; for, if we cut it, there will be only one set of actions paralyzed; while, by dividing a nerve which has a double origin,—viz. the fifth, we shall destroy two powers,—viz. voluntary motion and sensibility. We know, also, that, when we cut through the trunk of a nerve going to the hand, we destroy both sensibility and the power of motion.

"In reference to this subject, I shall state the result of certain experiments which were made about thirteen years ago, by Mr. Charles Bell.* The two sets of filaments by which each spinal nerve is connected to the spinal marrow were exposed: on irritating one set, convulsion of the muscles upon which the nerve was distributed ensued; but, when the other was excited, no perceptible effect was produced. These experiments we have often repeated, and always with the same results; but, from the violence necessarily used in making them, it has been difficult to ascertain which of the filaments bestows sensibility on the part. It was easily shown that, if only the posterior set was destroyed, the voluntary power over the muscles continued unimpaired; but the pain necessarily attendant upon the performance of the experiment prevented us from judging of the degree of sensibility remaining in the part."—*(From the Med.-Chirurg. Transactions.)*

"The experiments alluded to above have been often repeated, and always with the same results. I shall here copy verbatim the notes which I drew up on the 21st of March, 1821, as memoranda of the result of some experiments made with Mr. Bell upon that day, to be inserted in his private journal.

"But this part of the research (viz. certain experiments on the ninth and glosso-pharyngeal nerves,) was hurried in order to examine the spinal marrow. The bones of the spine were exposed at the lower part of the neck, by cutting through the muscles, &c. as quickly as possible; about two inches of the canal were exposed, by sawing through the crura of the spinous processes. The proper sheath of the spinal marrow was not opened; but, the whole being pulled a little to one side, the nerves were seen going off. On irritating the posterior origins, no convulsion was produced; neither was there any on pinching the ganglion: but, on pinching the anterior roots, or on pinching the two sets of origins at the same moment, a convulsion took place in the corresponding muscles. This was repeated on several of the nerves, and in all with the same results. Several of the pupils, who were assisting, were satisfied of there being a marked difference between the two sets of fibres: indeed, it appeared to them quite satisfactory that there was no convulsion produced by irritating the posterior roots of the ganglion."—*(London Medical and Physical Journal,* Oct. 1822.)

---

* Described in the Essay from which the preceding quotation is taken.

The paper from which this extract is taken, was read, and publicly discussed, in the Medico-Chirurgical Society, in April 1822, and was published in June 1822; M. Magendie's first experiments were made in July 1822. It is but justice to add, that M. Magendie did, in his Journal for October 1821, copy the description of the new views on the nervous system from Mr. Shaw's "Manual of Anatomy," where direct reference is made to the above experiments. This fact but ill accords with the statement made by M. Magendie, in his Journal for August 1822: "For a long time I have been desirous of discovering what effects would be produced upon an animal by dividing the posterior roots of the spinal nerves."—"I had not entertained the slightest idea of what would be the probable result of this experiment," &c.

But, laying aside all question of M. Magendie's having had a previous knowledge of the experiments, here is the admission that they were not founded on the views of anatomy, but that they were *mere* experiments: and, that he is not even *now* master of the question, is shown by hi shaving omitted all mention of the fifth pair, which has been proved by anatomy, and by experiments, to be in every respect similar to the nerves which rise by double roots from the spinal marrow.*

To those who are acquainted with investigations of this nature, it cannot be matter of surprise that M. Magendie should confess that he has been unable to draw any positive deductions from his experiments on different parts of the brain. When these are not founded on anatomy, physiology makes no advance, but vacillates backwards and forwards,—like the birds described in the Memoir. This is the old way of making experiments in the dark, although the Essays upon them are sure to be preceded or followed by something about Bacon, as if the experimentalists were following his steps, and were in the high way of philosophy.

* See the Number of this Journal for October 1822.

